# Single Coronary Artery in the Context of Vascular Disease: Anatomy, Development, Multimodality Imaging, and Clinical Interpretation

**DOI:** 10.3390/jcm15166435

**Published:** 2026-08-20

**Authors:** Musa Muhtaroglu, Hasan Birtan

**Affiliations:** 1 Department of Anatomy, Faculty of Medicine, European University of Lefke, Lefke 99728, Northern Cyprus, TR-10 Mersin, Türkiye; 2 Department of Cardiovascular Surgery, Dr. Burhan Nalbantoğlu State Hospital, Nicosia 99010, Cyprus; birtandr@yahoo.co.uk

**Keywords:** single coronary artery, coronary artery anomalies, coronary computed tomography angiography, anomalous coronary artery, clinical interpretation, vascular disease, multimodality imaging

## Abstract

**Background:** Single coronary artery (SCA) is a rare and anatomically heterogeneous congenital coronary anomaly in which the entire coronary circulation arises from a single aortic ostium. Its clinical significance depends on the ostial and proximal morphology, coronary course, associated coronary disease, symptoms and, where indicated, functional findings. **Methods:** PubMed/MEDLINE was searched with free-text terms from 24 December 2025 to 12 July 2026 and rerun on 9 August 2026; relevant reference lists were screened manually. **Results:** SCA-specific evidence consisted mainly of case reports, small series, and descriptive imaging studies. A single ostium or Lipton code does not fully describe the relevant anatomy. Reports should state whether a true common trunk is present, map the course and supplied territory of each major branch, and document proximal morphology and acquired coronary disease. Coronary computed tomography angiography (CCTA) is central to anatomical assessment in many adults. Functional testing is most informative when the method addresses the suspected mechanism. **Conclusions:** SCA should be interpreted by integrating detailed anatomy, symptoms, coexisting coronary disease and, when appropriate, mechanism-matched functional findings. The three-domain structure used here supports reporting and multidisciplinary discussion, but it is not a validated risk model or treatment algorithm. Prospective evidence on SCA-specific outcomes and management remains limited.

## 1. Introduction

Coronary artery anomalies encompass congenital variations in the origin, course, or termination of epicardial coronary arteries [1,2]. Their clinical significance stems not only from the name of the anomaly but also from its ostial and proximal morphology, its relationship to the great vessels, associated coronary disease, and the patient-specific clinical context [2,3]. Therefore, modern assessment is based on detailed anatomical characterisation and clinical correlation rather than a categorical label.

Within this spectrum, single coronary artery (SCA) is a rare, heterogeneous anomaly in which the entire coronary circulation arises from one aortic ostium, and it may follow different branching patterns and proximal courses [4,5]. The reported prevalence varies according to study design, population, and imaging method; for example, Lipton et al. reported 0.024% [6], whereas Desmet et al. found 33 cases among 50,000 angiograms (0.066%) [7].

The clinical interpretation of SCA cannot be completed simply by demonstrating a single ostium. SCA-focused publications support the combined assessment of proximal course, ostial morphology, and symptom context [4,8,9]. A significant proportion of haemodynamic inferences regarding interarterial or intramural anatomy derives from studies of anomalous aortic origin of a coronary artery (AAOCA) [3,10,11], while evidence on sudden events comes largely from the wider coronary anomaly literature [12]. Some publications use the term anomalous coronary artery from the opposite sinus (ACAOS); these inferences should not be applied directly as an SCA-specific risk rule. Concomitant coronary disease is documented in SCA series and cases, while procedural evidence includes broader anomalous coronary cohorts [13,14,15].

Coronary computed tomography angiography (CCTA) has advanced the three-dimensional characterisation of coronary anomalies by enabling assessment of the origin, proximal course, relationship to adjacent great vessels, and associated luminal or vessel wall disease within the same examination [16,17,18]. Risk data regarding sudden cardiac events are based largely on the broader coronary anomaly literature rather than SCA cohorts [12,19]. Most SCA-specific evidence consists of case reports, small series, and descriptive imaging studies [4,13,20]. Prospective management frameworks are better developed for AAOCA and should not be presented as direct SCA evidence [21,22]. In adults, concomitant atherosclerotic disease further complicates the attribution of symptoms to anomalous anatomy, acquired disease, or both [13,14].

This review synthesises the developmental basis, anatomical spectrum, imaging-based assessment, and clinical significance of SCA within the context of vascular disease. However, this study is not a systematic review or meta-analysis. SCA-specific publications have been prioritised and, where direct evidence is insufficient, sources from the AAOCA and broader coronary anomaly literature are used only to provide a mechanistic, imaging, or management context. Here, vascular disease refers primarily to concomitant atherosclerotic coronary artery disease and the systemic vascular risk profile that shapes symptom attribution and management in adults; it does not imply that SCA itself is acquired.

This review has two linked contributions. First, it considers SCA together with acquired coronary disease and the practical demands of adult interventional and surgical care. Second, it distinguishes findings reported directly regarding SCA from inferences drawn from AAOCA and the wider coronary anomaly literature. Anatomy, functional or haemodynamic findings, and the patient’s clinical vascular context are used as three reporting domains. They organise the evidence and support multidisciplinary discussion; they do not constitute a new or validated clinical model.

### Literature Search and Review Scope

This narrative review examined the literature on SCA development, anatomy, imaging, adult vascular disease, and clinical or procedural interpretation. A PubMed/MEDLINE search was conducted from 24 December 2025 to 12 July 2026 and rerun on 9 August 2026. PubMed Central and publisher websites were used to retrieve full texts, and the reference lists of relevant publications were screened manually. No lower publication year limit was applied, enabling classical classification and embryology studies to be considered alongside contemporary evidence.

The main free-text terms were ‘single coronary artery’, ‘single coronary ostium’, ‘coronary artery anomaly’, ‘anomalous aortic origin of a coronary artery’, ‘AAOCA’, ‘ACAOS’, ‘coronary computed tomography angiography’, ‘CCTA’, ‘intramural course’, ‘interarterial course’, ‘vascular disease’, ‘atherosclerosis’, ‘coronary imaging’, ‘fractional flow reserve’, ‘intravascular ultrasound’, ‘percutaneous coronary intervention’, and ‘surgery’. The principal concept combinations paired ‘single coronary artery’ or ‘single coronary ostium’ with terms for (i) CCTA, coronary imaging, intramural, or interarterial anatomy; (ii) atherosclerosis or vascular disease; (iii) fractional flow reserve or intravascular ultrasound; and (iv) percutaneous coronary intervention or surgery. Given that the searches were iterative, these groupings describe the principal combinations used rather than one prospectively archived Boolean string. Medical Subject Headings (MeSH) were not used as a predefined search strategy, and complete query strings were not archived prospectively.

Eligible sources included original studies, case series, clinically informative case reports, imaging studies, reviews, and guidance or consensus documents with sufficient English-language information to assess the relevant claim. Non-English reports, duplicate records, out-of-scope publications, conference abstracts without adequate clinical or methodological detail, and editorials without original data were excluded. When full text was unavailable, a record was used only if its English abstract and bibliographic information were sufficient to verify a narrow factual statement; it was not used for detailed methodological or outcome inference.

SCA-specific publications were preferred for prevalence, classification, anatomy, imaging, and procedural questions. Wider AAOCA evidence was included only when direct SCA evidence was insufficient and the study addressed a comparable proximal morphology, physiological mechanism, imaging measurement, or management question. This evidence is identified as AAOCA-derived context rather than presented as direct SCA evidence.

M.M. conducted the literature search and initial relevance assessment; both authors reviewed the clinical and surgical use of the retained sources in the manuscript. Records were not screened independently by both authors, so no formal disagreement resolution procedure was applicable. Duplicate records were consolidated by title, authorship, and digital object identifier, where available. Given that a prospective screening log was not established, title–abstract and full-text screening counts were not reconstructed retrospectively. The final narrative synthesis cites 85 publications. No Preferred Reporting Items for Systematic Reviews and Meta-Analyses (PRISMA)-based selection, quantitative pooling, or formal risk-of-bias scoring was undertaken. Supplementary Table S1 summarises the principal SCA-specific studies and their evidence boundaries.

During the preparation of this manuscript, OpenAI’s ChatGPT in a GPT-5-based Codex environment was used to support English-language drafting and refinement, terminology consistency, reference checking, and the programmatic preparation of original schematic figures. It was not used to perform statistical or other data analysis or to make autonomous clinical judgements. The authors have reviewed and edited all outputs and take full responsibility for literature selection, anatomical accuracy, clinical interpretation, and the content of the manuscript.

## 2. Developmental Basis of Single Coronary Artery

The developmental basis of SCA should be assessed within the framework of the multi-stage and dynamic embryological organisation of the coronary artery system [23,24]. Currently, the coronary vascular system is understood to reflect a complex network structure resulting from diverse cellular contributions and remodelling processes, rather than a simple structure arising from a single origin [23,24,25]. Epicardial cells, subepicardial mesenchyme, and endothelial precursors all contribute to this organisation, whilst connections established with the aortic root play a critical role in determining the final architecture of the system [24,26,27]. Thus, the final anatomical arrangement of the coronary arteries should be understood as a developmental process dependent not only on vascularisation but also on the selective establishment of connections, the regression of certain segments, and the dominance of others [23,26,27].

In normal development, the right and left coronary arteries form a two-ostium system as a result of the separate connections they establish with the aortic sinuses [25,26,27]. However, variations in this connection process may affect the number of ostia, their location, and the subsequent branching architecture. In this context, SCA can be regarded as a rare developmental variation in which the entire epicardial coronary circulation is maintained via a single ostial connection [5,23,24]. Nonetheless, no single, definitive embryological mechanism has been identified to explain all subtypes of SCA. Given that coronary development is mosaic and involves multiple cellular and remodelling pathways, more than one developmental route to an SCA phenotype is biologically plausible [23,24].

The anatomical heterogeneity of SCA is consistent with the view that different developmental pathways can lead to a similar single-ostium phenotype. In some cases, contralateral coronary distribution arises through distal continuity; in others, unusual proximal courses reaching different regions of the myocardium can be observed. CCTA-based cases and small series demonstrate this morphological diversity [4,5,28].

Although SCA is congenital, its embryological origin does not determine clinical significance in adulthood by itself [5,23]. Embryology provides a framework for understanding the formation of the anomaly, whereas adult interpretation depends on the resulting anatomy, accompanying atherosclerotic disease and, where necessary, functional findings [13,14,23].

## 3. Anatomical Spectrum and Classification

Beyond the single-ostium definition, SCA reports should specify the sinus of origin, the branching pattern, the formation of the contralateral coronary bed, and the proximal course [4,5,7]. Feature-level CCTA analysis in left-sided ACAOS also illustrates the value of describing proximal morphology, although that evidence is not SCA-specific [29].

Several related terms describe different anatomical features. A single coronary ostium means that the coronary circulation has one aortic opening. A common trunk is a measurable proximal segment before the major branches separate. Single coronary artery remains the traditional umbrella term used in the Lipton literature. Traditional SCA classification includes distal continuity patterns [6], and contemporary nomenclature reserves single-trunk AAOCA (ST-AAOCA) for a true shared aortic trunk that gives rise to all major coronary branches [30]. Accordingly, a single ostium with immediate branching or bifurcation within the aortic wall may fit traditional SCA usage without meeting the ST-AAOCA definition [6,30].

Before SCA is classified, a truly single-aortic ostium should be confirmed by tracing the origin and continuity of all major epicardial branches. Left-main ostial atresia or agenesis can mimic SCA, particularly when distal filling occurs through an intercoronary communication; an agenesis-related classification dilemma is illustrated in a published case [31]. Anomalous left coronary artery from the pulmonary artery (ALCAPA) and a fistula-dependent pattern of coronary filling are separate possibilities that should also be excluded [32]. CCTA can resolve the origin and three-dimensional continuity, while invasive angiography may be required when retrograde filling obscures the underlying anatomy [31].

The Lipton classification provides a common language for SCA [6,33]. Its code has three parts: the right (R) or left (L) sinus of origin, distribution group (I–III), and a suffix describing the proximal course. Group I denotes distal continuity. In Group II, an anomalous contralateral branch arises from the proximal main vessel. Group III describes separate left anterior descending (LAD) and left circumflex (LCx) branches arising from the proximal right coronary artery (RCA) [6,20].

The classic Lipton course suffixes are A (anterior/prepulmonic), B (interarterial, between the great vessels), and P (posterior/retroaortic) [6]. In the Yamanaka–Hobbs modification, S denotes a septal/transseptal course, and C denotes a combination of directional courses [20,33]. C should not be interpreted as an independent or validated risk category; in complex anatomy, the course of each branch should be specified.

**Figure 1:**
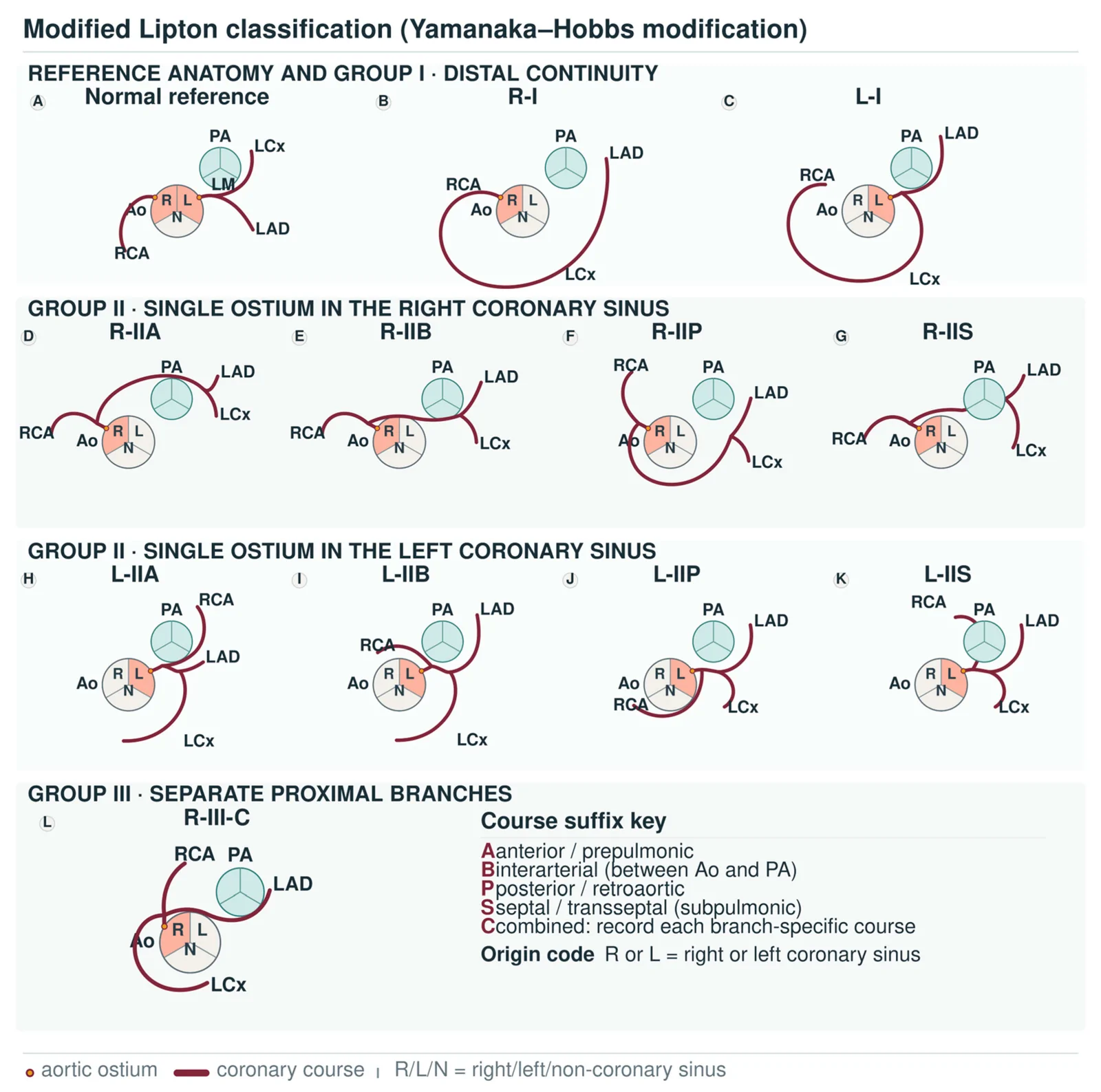
CCTA-oriented, caudal-to-cranial schematic of the modified Lipton classification (Yamanaka–Hobbs modification) of a single coronary artery. (**A**) Normal two-ostium reference anatomy. Group I: (**B**) R-I and (**C**) L-I distal continuity into the contralateral territory. Group II: Right-sinus configurations (**D**–**G**, R-IIA/B/P/S) and left-sinus configurations (**H**–**K**, L-IIA/B/P/S), with A denoting anterior/prepulmonic, B interarterial, P posterior/retroaortic, and S septal/transseptal course [6,20,33]. (**L**) Representative R-III-C anatomy, in which the LAD and LCx arise separately from the proximal RCA and follow LAD-B and LCx-P courses. Given that C represents branch-specific combined anatomy, panel (**L**) is illustrative rather than exhaustive. Panels (**B**–**L**) contain one ostium in the highlighted aortic sector; panel (**A**) shows two separate ostia. The codes describe anatomy, not clinical risk or treatment [30]. Color coding: orange shading indicates the coronary sinus containing the ostium (both sinuses in panel (**A**)), orange dots mark aortic ostia, burgundy lines show coronary courses, and pale blue–green denotes the pulmonary artery. Ao, aorta; CCTA, coronary computed tomography angiography; LAD, left anterior descending artery; LCx, left circumflex artery; LM, left main coronary artery; N, non-coronary sinus; PA, pulmonary artery; R/L, right/left coronary sinus; RCA, right coronary artery.

The Lipton system classifies SCA by sinus of origin, distribution group, and proximal course. The Yamanaka–Hobbs modification extends the course descriptors by adding septal/transseptal (S) and combined (C) patterns. The Shirani–Roberts system provides a more detailed classification, based on the site of the solitary ostium and the route of the aberrant coronary artery [34,35]. This review uses the modified Lipton classification as its primary system because it is the most widely recognised in clinical reporting; the Shirani–Roberts terminology is used when it clarifies the anatomy described in that system. Figure 1 presents a CCTA-oriented, caudal-to-cranial schematic of normal reference anatomy, Groups I and II with A/B/P/S courses, and a representative R-III-C configuration (LAD-B/LCx-P); because C does not define one fixed geometry, this panel is illustrative rather than exhaustive [6,20,33].

Coronary dominance is conventionally defined by the artery that gives rise to the posterior descending artery (PDA). In SCA, that label provides limited information because all coronary inflow begins at one ostium. Reports should instead name the origin of the PDA and describe the posterolateral branches. Anatomical studies have shown that inferior-wall arterial supply and PDA number can vary [36,37].

Terminal-branch anatomy also matters. Dual LAD anatomy has been reported with SCA [38], and its wider morphological range has been described using CCTA and post-mortem examination [39,40]. Duplicated PDA anatomy can likewise alter the distribution of inferior and septal supply [37]. Thus, reports should document branch number, calibre, length, course, and supplied territory, particularly when percutaneous coronary intervention (PCI) or surgery is planned.

The course label provides an anatomical description; by itself, it does not determine outcome, prognosis, or treatment. In interarterial or potentially intramural anatomy, ostial morphology, take-off angle, proximal narrowing, and the relationship to the great vessels should also be assessed [3,11,17]. Conversely, anterior, retroaortic, or septal courses should not be automatically labelled ‘benign’ without considering the complete anatomy and clinical context.

**Figure 2:**
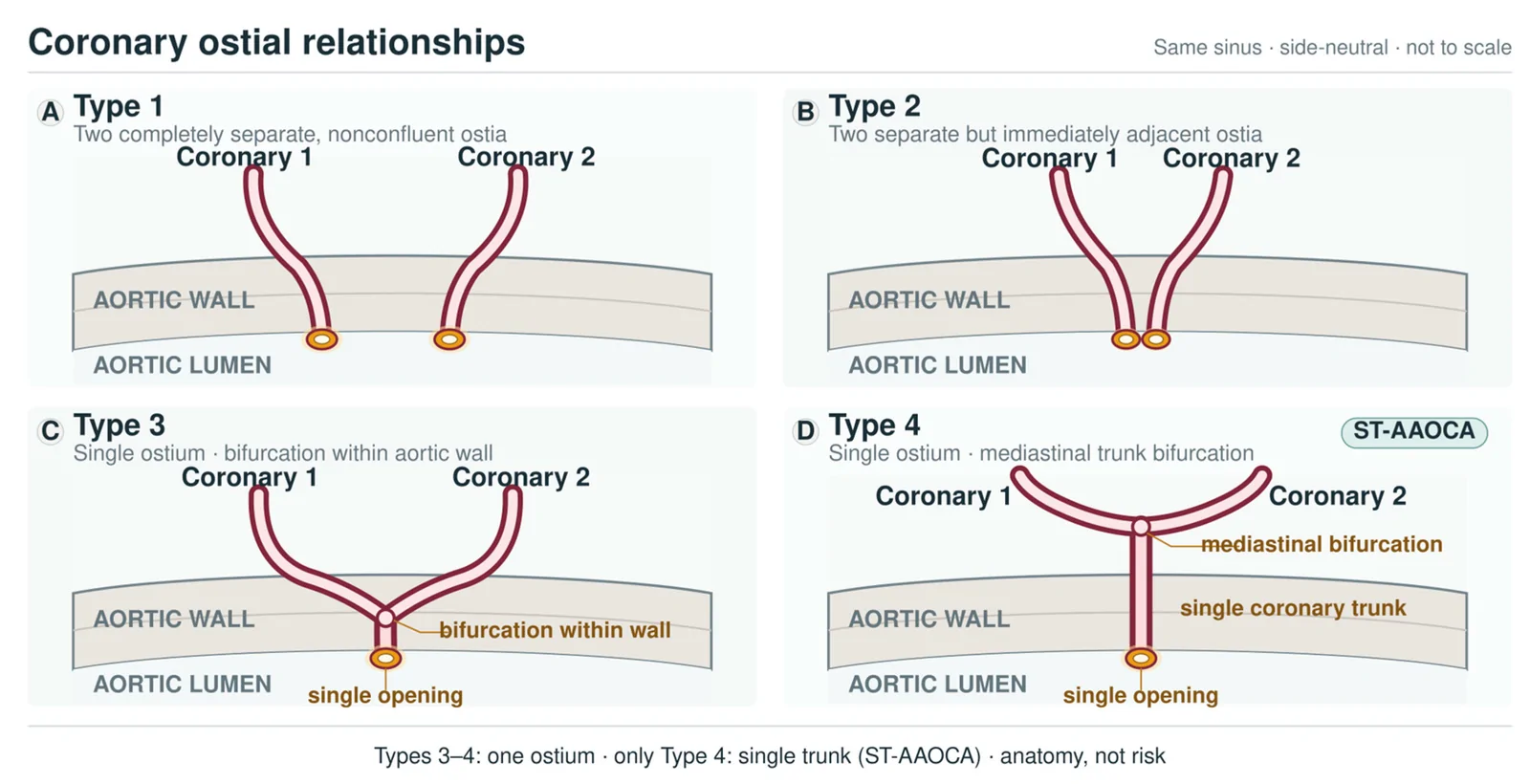
Schematic cross-sectional representation of the four-type relationship between coronary ostia arising from the same aortic sinus: (**A**) Type 1, two completely separate, nonconfluent ostia; (**B**) Type 2, two separate but immediately adjacent ostia; (**C**) Type 3, a single ostium with bifurcation into separate vessels within the aortic wall; and (**D**) Type 4, a single coronary trunk that continues beyond the aortic wall before bifurcating into separate vessels in the mediastinum [30]. Types 3 and 4 both have one ostium, but only Type 4 represents a single coronary trunk and therefore meets the ST-AAOCA nomenclature [30]. The diagram is side-neutral and not to scale, and it depicts the ostial relationship rather than risk or treatment. AAOCA, anomalous aortic origin of a coronary artery; ST-AAOCA, single-trunk anomalous aortic origin of a coronary artery.

Table 1 links the modified Lipton classification to contemporary CCTA reporting. The Lipton code describes the sinus of origin, distribution group, and proximal course, whereas ST-AAOCA describes the presence of a true shared trunk. These systems answer different anatomical questions and should not be treated as interchangeable. Therefore, Figure 2 isolates the four-type ostial relationship classification; that is, Types 1 and 2 have two ostia, whereas Types 3 and 4 have one. Only Type 4 has a single coronary trunk that continues beyond the aortic wall before bifurcating in the mediastinum and therefore meets the ST-AAOCA terminology. Accordingly, one ostium alone does not justify the ST label, and the ostial relationship scheme complements rather than replaces the modified Lipton classification [6,30].

In adults, CCTA can depict the ostium, any proximal trunk, the branch-level course, relationships to the great vessels, and coexisting coronary disease in three dimensions [5,18,41]. Studies of SCA CCTA cohorts have further shown that proximal dimensions and course can vary between patients [42,43]. Reports should therefore describe the anatomy, rather than relying on the Lipton code alone.

Acquired pathology, such as spontaneous coronary artery dissection in an SCA case, should be recorded separately from the congenital classification [44]. The case literature includes interarterial patterns [45,46], while other reports have described a transseptal left main course [47] and the absence of a contralateral ostium [48]. Another report illustrated a right-sided SCA configuration [49]. Virtual endoluminal CCTA views may add detail at the aortic root and proximal coronary segment [50].

**Table 1:** Classification and CCTA-oriented morphological reporting framework for single coronary artery.

Framework Element	Definition and Representative Coding	CCTA-Oriented Reporting Focus	Interpretive Boundary	Evidence Category and Support
Definition, true single ostium and common trunk	Traditional SCA usage denotes an entire epicardial coronary circulation arising from one aortic ostium. A common trunk requires a measurable shared proximal segment before major branches divide.	Confirm both aortic sinuses; exclude pulmonary-origin and fistula- or collateral-dependent mimics; and state whether branching occurs within the aortic wall, immediately beyond it, or after a measurable common trunk.	By itself, a single ostium does not prove a common trunk, define ST-AAOCA, or determine clinical significance.	Traditional SCA definition [6,7]. Left-main agenesis dilemma [31]. Pulmonary-origin and fistula differential context [32]. Component-based AAOCA terminology [30].
Distribution group I	R-I or L-I: One vessel continues distally to supply the contralateral coronary territory.	Describe distal continuity, PDA origin, posterolateral branches, vessel calibre, terminal-branch duplication, supplied territory, and luminal disease.	Usually a descriptive category; interpretation depends on course, symptoms, objective ischaemia, and acquired disease.	Classical classification [6]. Direct angiographic SCA evidence [7,51,52].
Distribution group II	R-II or L-II: An anomalous branch arises from the proximal main trunk and reaches the contralateral coronary distribution. A/B/P are classical course suffixes; S and C appear in the Yamanaka–Hobbs modification.	Report take-off site and angle, course relative to Ao/PA, proximal narrowing, suspected intramural segment, and branch-level anatomy.	Course and proximal morphology are more informative than the group label alone.	Classical Lipton and Yamanaka–Hobbs classification evidence [6,33].
Distribution group III	R-III: LAD and LCx arise separately from the proximal RCA or a right-sided common trunk; complex variants may have branch-specific mixed courses.	Map LAD and LCx separately, including their courses, supplied territories, calibre, and procedural implications.	Complex patterns should be reported branch by branch rather than compressed into a single clinical risk label.	Classical classification [6]. Direct SCA report with dual LAD [38].
A suffix	Anterior/prepulmonic course; representative coding R-IIA or L-IIA.	Confirm the anterior relationship to PA and document ostial/proximal geometry.	Anterior/prepulmonic course should be described anatomically; its clinical relevance remains dependent on patient-specific morphology and context.	Classical classification evidence [6,33].
B suffix	Interarterial course between the great vessels; representative coding R-IIB or L-IIB.	Assess the Ao–PA relationship, slit-like ostium, acute take-off angle, proximal narrowing, intramural segment, and suspected dynamic compression.	Anatomy warranting heightened attention; interpretation should integrate detailed morphology, symptoms, and functional evidence when indicated.	Classical course description [6,33]. Proximal morphology interpretation is derived mainly from AAOCA evidence [3,11].
P suffix	Posterior/retroaortic course; representative coding R-IIP or L-IIP.	Describe retroaortic trajectory, relationship to the aortic root/valves, and coexisting aortic or coronary disease.	The retroaortic label is descriptive and should not be used alone to infer clinical risk.	Classical classification evidence [6,33].
S suffix	Septal/subpulmonic/transseptal course introduced in the Yamanaka–Hobbs modification; representative coding R-IIS or L-IIS.	Define the septal or subpulmonic pathway, any intramyocardial/intraseptal component, and relationship to PA/RV outflow.	S is an anatomical descriptor; clinical interpretation depends on patient-specific morphology, symptoms, and functional evidence.	Yamanaka–Hobbs modification and SCA descriptive evidence [20,33].
C suffix	Combined or mixed course involving more than one directional component in the Yamanaka–Hobbs modification.	State which branch follows which course (for example, LAD-B with LCx-P), rather than relying on C alone.	C is a descriptive modifier, not a validated or stand-alone risk category.	Yamanaka–Hobbs modification and SCA descriptive evidence [20,33].
Classification systems	Lipton combines sinus of origin, distribution group, and proximal course suffix; Yamanaka–Hobbs adds S and C; Shirani–Roberts classifies the site of the solitary ostium and route of the aberrant artery.	State the system used and provide a component-level anatomical description when one code does not capture the full pattern.	The modified Lipton system is used as the primary clinical language in this review; Shirani–Roberts terminology is complementary.	Classical primary classification sources [6,33,35].
Coronary dominance and inferior supply	Dominance is conventionally defined by the artery giving rise to the PDA.	Identify the origin of the PDA and the number and distribution of posterolateral branches.	Right- or left-dominance labels have limited discriminating value when the whole myocardium is supplied from one aortic origin.	Direct SCA CCTA series [41,42,43]. Contextual inferior-branch anatomy [36,37].
Terminal-branch duplication	Dual LAD or duplicated PDA patterns alter the distal perfusion map despite a common ostial origin.	Report branch number, calibre, length, course, and supplied myocardial territory.	Branch-level anatomy identifies the vessels that require protection during PCI or surgery.	Dual LAD reported with SCA [38]. Broader dual-LAD evidence [39,40] and double-PDA evidence [37].
CCTA morphology beyond classification	Classic labels do not fully capture ostial morphology, take-off angle, a suspected intramural segment, proximal narrowing, or Ao–PA relationships.	Use multiplanar and three-dimensional CCTA to describe the complete proximal course and adjacent great vessel relationships.	CCTA complements classification; morphology alone should not be converted into a treatment rule.	Direct SCA CCTA series [41,42,43]. Additional SCA imaging context [5]. Risk-agnostic AAOCA terminology [30].

Abbreviations: AAOCA, anomalous aortic origin of a coronary artery; Ao, aorta; CCTA, coronary computed tomography angiography; LAD, left anterior descending artery; LCx, left circumflex artery; PA, pulmonary artery; PDA, posterior descending artery; PCI, percutaneous coronary intervention; RCA, right coronary artery; R/L, right/left coronary sinus; RV, right ventricle; SCA, single coronary artery; ST-AAOCA, single-trunk anomalous aortic origin of a coronary artery. A/B/P are the classical Lipton course suffixes; S and C were added in the Yamanaka–Hobbs modification [6,33]. The table supports structured anatomical reporting and does not assign risk or treatment.

## 4. Pathophysiological and Haemodynamic Implications

The potential pathophysiological significance of SCA depends on the proximal vessel geometry, vessel course, ostial morphology, and associated coronary artery disease rather than on the single ostium itself [4,13]. SCA does not automatically imply haemodynamic impairment. Most proposed mechanisms of dynamic compression, ostial or proximal narrowing, and flow restriction derive from AAOCA studies [3,10,11], and should therefore be applied to SCA cautiously and in an anatomy-specific manner.

A severe lesion on the sole ostium or within a shared proximal trunk before the major branches diverge can jeopardise several myocardial territories and may function as a left-main-equivalent lesion [53]. The territory at risk depends on the position of the lesion relative to the branch points and the distal perfusion map. SCA provides no second independently originating aortic inflow. Collateral vessels may still be present, but reserve cannot be assumed to equal that of a two-ostium system; the location of a proximal lesion is therefore especially important.

An interarterial course requires detailed assessment, particularly when accompanied by an intramural segment, slit-like ostium, acute take-off angle, or proximal narrowing. In AAOCA studies, the interarterial position often accompanies, rather than fully explains, the relevant flow-limiting morphology [3,10,11]. In SCA, these observations should be integrated with age, symptoms, coexisting coronary disease and, where indicated, objective functional findings.

An intramural segment may influence the shape and compliance of the proximal lumen through its course within the aortic wall. No SCA-specific cohort has validated intramurality as an independent outcome predictor or automatic indication for intervention [20]. Its relevance should be assessed based on the ostium, proximal narrowing, and clinical context [3,11].

Haemodynamic interpretation should focus on the location of any narrowing relative to major branch points and the supplied myocardial territory, rather than on the ostium count alone. Although the theoretical importance of flow distribution in single-ostium circulation is recognised, SCA-specific haemodynamic evidence remains limited [4,8,20]. A morphology showing no significant obstruction at rest may behave differently under physiological stress; functional assessment can complement anatomical imaging in selected cases when its protocol addresses the suspected mechanism [8,54,55].

Coexisting coronary artery disease in adults makes it difficult to attribute symptoms and signs of ischaemia to anomalous anatomy [13,14,56]. Available SCA reports document the coexistence of SCA and atherosclerotic disease but do not establish causation [13,14]; broader anomalous coronary cohorts did not demonstrate increased significant or obstructive coronary disease attributable to anomalous origin [57,58]. Therefore, fixed stenosis, distal perfusion, anomalous proximal morphology, and the clinical context should be assessed separately.

## 5. Clinical Presentation and Heterogeneity of Clinical Significance

SCA presents a variable clinical spectrum, ranging from asymptomatic and incidentally detected cases to conditions that become significant during ischaemia assessment, acute coronary syndrome, and interventional or surgical planning [4,13,20]. With the widespread adoption of advanced imaging techniques, many cases with an unclear relationship to symptoms are identified during investigations carried out for other reasons [5,9]. For this reason, SCA should not be reduced to a simple ‘benign’ or ‘malignant’ dichotomy.

In symptomatic cases, chest pain, exertion-related complaints, dyspnoea, palpitations, presyncope, or syncope may be reported [9,13,52]. Nevertheless, these symptoms cannot be attributed solely to anomalous coronary anatomy. In adults, atherosclerotic disease, structural heart disease, arrhythmia, or ischaemia with non-obstructive coronary arteries (INOCA) may be alternative or coexisting explanations [14,56]. General adult coronary anomaly series also show that cases are frequently detected during investigations for symptoms or coronary disease [58,59].

Clinical significance cannot be determined from the sinus of origin or Lipton label alone. Proximal course, relationship to the great vessels, suspected intramural segment, ostial morphology, objective ischaemia, symptom profile, and concomitant atherosclerotic disease should be correlated [3,4,13]. Structural findings and physiological measurements may be discordant [55,60,61].

Age and activity level are contextual variables that influence clinical interpretation. Whilst exercise-related symptoms and events may be predominant in younger patients, atherosclerotic coronary artery disease and systemic vascular risk factors become more prominent in adults [12,13,19]. As no age-based threshold has been validated specifically for SCA, age alone should not be used to determine clinical significance or management [20].

Incidental detection does not necessarily imply clinical insignificance, nor does the presence of symptoms establish a causal link to the anomalous anatomy.

## 6. Imaging-Based Evaluation and Diagnostic Strategy

Imaging must define the single ostium, branching pattern, formation of the contralateral coronary bed, and relationships to the great vessels rather than merely confirm SCA [5,18,62]. It therefore underpins morphological description and subsequent clinical interpretation [5,18].

Imaging assessment distinguishes anatomical characterisation from functional interpretation. Anatomical imaging defines the sinus of origin, branching, and course, whereas functional assessment examines myocardial perfusion or physiological behaviour under stress. These levels should be integrated, but testing should proceed stepwise according to the clinical question [18,54].

Transthoracic echocardiography may help to identify the coronary ostia, particularly in children and in the care of experienced hands. In adults, acoustic window limitations and incomplete visualisation of the coronary course mean that echocardiography rarely suffices for complex SCA mapping; it remains an initial or complementary method [18,63].

Conventional invasive coronary angiography is valuable for assessing the lumen, coexisting stenosis, and interventional anatomy [16,18]. Its two-dimensional projections may not fully show complex three-dimensional courses or relationships to the great vessels. In adults with uncertain complex anatomy, CCTA commonly provides the required spatial definition [17,18].

CCTA is central to detailed anatomical characterisation in many adults with SCA [4,5,18]. It can show the ostial morphology, proximal course, relationships to the great vessels, and a suspected intramural segment [3,11,17]. However, anatomical appearance alone does not establish functional significance [3,54].

A structured CCTA report should state the exact sinus and ostial location, the number of ostia, the presence or absence of a measurable common trunk, branching patterns, and the course of each major branch [30,41,42]. It should also map PDA origin and terminal branches [37], record plaque or stenosis, and identify implications for catheter engagement or revascularisation [64]. Ostial shape, take-off angle, proximal calibre, and suspected intramurality should be reported as separate observations rather than compressed into a single risk label.

CTA measurements are not fully standardised across studies. In AAOCA cohorts, inter-reader reliability varies by feature, and comparisons with surgical measurements show that accuracy is feature-dependent; nitroglycerin use may also affect distal coronary dimensions [65,66]. Reports should therefore document the measurement method and relevant acquisition conditions, and these AAOCA reproducibility data should not be assumed to apply directly to SCA.

CCTA is central to anatomical assessment in many adults, but it is not the best test for every patient. Renal function, iodinated contrast hypersensitivity, radiation exposure, heart rate and rhythm, and severe coronary calcification should be considered because these factors may affect patient selection, acquisition, image quality, or interpretability [18,67]. Evidence linking CCTA findings to management decisions in broader coronary anomaly populations remains preliminary [68].

Three-dimensional CCTA adds ostial, proximal, and distal detail beyond the angiographic silhouette underlying the Lipton classification. Cases with the same code may differ materially in their spatial relationships, reinforcing the need to report morphology alongside classification [5,6,17].

Cardiac magnetic resonance imaging (CMR) is complementary when ventricular function, tissue characteristics, perfusion, or ischaemia require assessment. It avoids ionising radiation and provides structural and myocardial information, but generally has a lower spatial resolution than CCTA for fine coronary anatomy. CMR is therefore usually used in a supplementary manner, rather than as the primary method for detailed SCA mapping [16,18].

Functional tests should be chosen for the physiological question they are expected to answer. Exercise electrocardiography (ECG), stress echocardiography, nuclear perfusion imaging, and stress CMR measure different consequences of exertion or ischaemia and are not interchangeable [3,54]. An abnormal result may reflect fixed coronary disease or a non-obstructive mechanism rather than the anomalous anatomy [56]. Direct SCA evidence is limited to case reports and a small case series [8,45,55]; comparative physiological evidence comes mainly from AAOCA cohorts [54].

Intravascular ultrasound (IVUS) provides cross-sectional assessment of the ostium and proximal lumen and is the practical invasive reference method for directly depicting lateral or dynamic proximal lumen compression. In direct SCA evidence, selected patients in a five-patient case series underwent instantaneous wave-free ratio assessment and IVUS at rest and during pharmacological stress [45], while IVUS and fractional flow reserve (FFR) were assessed during deep breathing in an individual case [55]. In a prospective cohort of adults with anomalous aortic origin of the right coronary artery (R-AAOCA), resting IVUS measurements were compared with dobutamine-based FFR rather than treated as interchangeable tests. Adenosine FFR primarily tests hyperaemic pressure loss, whereas a dobutamine–atropine–volume challenge is intended to reproduce an exercise-like dynamic mechanism and is more demanding [69]. A normal vasodilator FFR may therefore fail to exclude a dynamic abnormality [69]. CT-derived fractional flow reserve (FFRCT) has been applied directly in an SCA case and studied in broader AAOCA cohorts [8,70,71], but no SCA-specific threshold has been validated.

A technically sound negative study is most reassuring when the test reproduces the suspected mechanism and agrees with the anatomy and clinical picture. A single negative result should not automatically dismiss a notable proximal feature, but anatomy should not automatically outweigh repeatedly negative, technically appropriate physiological findings. In the adult AAOCA MuSCAT (Multicenter Study on Coronary Anomalies in The Netherlands) cohort, non-invasive and invasive functional results were discordant in 28% of patients, and management recommendations changed in 20% of the cohort; these findings illustrate uncertainty but are not SCA-specific outcome evidence [60].

In adults, imaging must assess congenital anatomy and acquired coronary disease within the same evaluation [13,17]. CCTA can depict both anomalous morphology and luminal disease [5,17], whereas invasive angiography can clarify a lesion and guide intervention [15]. The diagnostic strategy should determine whether SCA is isolated or coexists with clinically relevant coronary disease. Table 2 summarises the practical roles of the principal imaging modalities.

## 7. Single Coronary Artery in Adult Vascular and Procedural Context

SCA is a congenital anomaly; nevertheless, in adult clinical practice, it is assessed within the same context as acquired coronary artery disease and procedural requirements [13,14,53]. This overlap makes it difficult to interpret the cause of symptoms and the clinical significance of the anomaly solely based on its anatomical classification.

In adults, chest pain, dyspnoea or signs of ischaemia may arise from an anomalous proximal course, atherosclerotic stenosis or another non-obstructive mechanism [13,14,56]. Therefore, anomalous anatomy should not automatically be accepted as the cause of symptoms; luminal disease and alternative ischaemic mechanisms must also be assessed.

The relationship between SCA and atherosclerotic disease should be described as coexistence unless causality is demonstrated. SCA reports document coexistence and procedural relevance [13,14], whereas broader anomalous coronary cohorts found no overall excess of significant or obstructive disease attributable to anomalous origin [57,58]. Given that those comparative data are not SCA-specific, they neither establish nor exclude a phenotype-specific association. Isolated acquired vascular events should not be interpreted as evidence of causality or increased susceptibility.

For each scenario, the priority is to define the anatomical or physiological information that can resolve the immediate question, while keeping alternative explanations and procedural hazards visible. Table 3 summarises representative adult SCA scenarios, their priority questions, and the applicable evidence boundaries.

Across these scenarios, acquired vascular risk influences overall cardiovascular risk and interpretation; it does not create an SCA-specific risk category. Direct SCA evidence documents coexistence of SCA and coronary disease [13], while a broader coronary anomaly CCTA cohort provides indirect comparative context [58]. Procedural relevance is illustrated by SCA cases involving valve disease or proximal stenosis [14,53]. Taken together, these sources do not establish a causal relationship between SCA and atherosclerosis [13,14,53,57,58].

## 8. Structured Reporting and Interpretive Boundaries

No SCA-specific cohort has established a consistent relationship between any single anatomical feature and clinical outcomes [20]. Associations reported in the broader coronary anomaly literature [54] cannot be transferred uniformly to all SCA phenotypes. Rather than proposing a new risk model, this review structures the three following areas of information already used in the literature within the context of SCA: detailed anatomy, functional or haemodynamic evidence, and the patient-level clinical–vascular context.

The anatomical domain encompasses ostial origin, branching pattern, proximal course, ostial morphology, take-off angle, proximal narrowing, suspected intramural segment, and relationship to the great vessels [3,11,17]. These findings define the structural substrate, but do not alone constitute a definitive indicator of outcome or treatment for SCA.

The functional or haemodynamic domain records whether a suspected anatomical mechanism is accompanied by objective ischaemia, stress-induced symptoms or an abnormality on an appropriately selected physiological test. Interpretation remains conditional on the protocol, technical quality, and agreement with the anatomical and clinical domains [8,54,55].

The clinical–vascular context encompasses symptoms, syncope, activity profile, age, concomitant coronary artery disease, and general cardiovascular comorbidities [3,13,19]. This information informs the patient-specific interpretation of anatomical or functional findings.

Concordance among these domains can strengthen a causal interpretation, whereas discordance identifies unresolved uncertainty. Table 3 includes this relationship within the relevant adult scenarios. No prospective study has validated the three-domain structure for prediction or treatment selection in SCA.

## 9. Management Implications and Decision-Making Considerations

Management should be individualised according to detailed anatomy, symptoms, objective functional findings, concomitant coronary artery disease, and procedural objectives [3,13,14]. Morphological heterogeneity means that the same classification label need not lead to the same management decision.

Controlled SCA-specific management studies are limited [20]. Direct SCA evidence comes mainly from case reports and small procedural series [64,72,75]. Most decision pathways and formal guidance concern AAOCA [3,21,22]. These sources should not be treated as equivalent or used as an automatic SCA decision rule.

When anatomy, symptoms, and physiological findings are discordant, further evaluation should address the unresolved question rather than repeat testing without a defined purpose. Selected complex cases may benefit from multidisciplinary review, but the available evidence does not support an SCA-specific treatment algorithm.

Imaging supports but does not independently determine management. CCTA defines proximal, ostial, intramural, luminal and procedural anatomy, while selected physiological tests assess functional correlates [17,18,54]. These findings should be interpreted jointly with symptoms and patient-specific clinical goals [22,32].

Direct SCA PCI reports emphasise selective cannulation, guide support, branch mapping, and protection of the sole ostium [64,72]. A broader anomalous coronary PCI cohort provides procedural context but is not SCA-specific [15]. A lesion at the sole ostium or shared trunk requires particular care, as catheter trauma or dissection may jeopardise several territories.

Operations developed for isolated AAOCA cannot be transferred automatically to SCA. Unroofing requires an appropriate intramural segment and careful assessment of the commissure and adjacent branches. Reimplantation requires mobilisation of the proximal anomalous artery and care to avoid vessel trauma or kinking; ostial reconstruction with patch material may be required when direct aortic anastomosis is unsuitable [3]. AAOCA surgical series provide indirect outcome context for these approaches [76,77,78]. Bypass grafting may be compromised by competitive native flow when no fixed stenosis is present [3]. During valve or aortic root surgery, the sole ostium and the course of every dependent branch should be mapped and protected [14,75]. These principles are derived from the AAOCA series and SCA case reports, not comparative SCA trials.

No SCA-specific comparative evidence defines universal exercise restriction or return-to-activity criteria. Decisions should consider exertional symptoms, detailed anatomy, results from an appropriate stress protocol, any treatment performed, and the intended level of activity. AAOCA consensus guidance and observational evidence may inform discussion, but should not be applied wholesale to every SCA pattern [73,79,80].

## 10. Future Directions and Knowledge Gaps

Despite advances in anatomical characterisation, SCA-specific data remain insufficient to standardise long-term prognosis, functional significance, or management decisions [4,13,20].

The primary priority is to establish multicentre prospective cohorts linking anatomical subtypes to long-term outcomes. Future studies should standardise the recording of the Lipton classification, ostial morphology, take-off angle, proximal narrowing, intramural segment, branch-level course, and concomitant atherosclerotic disease [6,11,17].

A second key area is the relationship between anatomy and physiological significance. Studies comparing CCTA measurements with stress imaging or invasive physiology have progressed particularly in anomalous aortic origin of the right coronary artery (R-AAOCA) [54,69,81]; these findings do not constitute a validated decision rule for SCA. SCA-specific studies should combine anatomical, functional, and long-term clinical data within the same protocol. The NARCO (Noninvasive Anatomical Assessment for Ruling out Hemodynamically Relevant Coronary Artery Anomalies in Adults) study design illustrates prospective comparison of CCTA with invasive assessment in R-AAOCA, not SCA [82].

The third knowledge gap concerns the interaction between SCA and acquired vascular disease. SCA-specific evidence does not establish SCA as an independent predictor of atherosclerosis [13,14,20], and broader anomalous coronary cohorts found no increased significant or obstructive disease attributable to anomalous origin [57,58]. In adults, however, coexisting single coronary anatomy and coronary disease may alter symptom interpretation, revascularisation planning, and procedural risk [13,14,53]. This relationship must be assessed in conjunction with age, plaque burden, and lesion location.

Standardisation of terminology and reporting is also a priority. In addition to the classification code, ostial and proximal morphology, relationships to the great vessels, suspected intramural segment, concomitant stenosis, and functional findings should be reported clearly [17,18]. Automated CCTA analysis has shown feasibility for detecting and classifying AAOCA [83]; however, its application to SCA requires separate validation and expert clinical interpretation.

Comparative data specific to SCA are also required for interventional and surgical planning. Direct SCA evidence remains limited to PCI and cardiac surgery reports or small series [64,72,75]. Patient-specific three-dimensional planning has been described in a broader anomalous coronary PCI case [84], while surgical outcome series concern AAOCA [78,85]. These sources illustrate technical issues but do not establish comparative SCA treatment.

### Evidence Boundaries and Limitations of the Review

This review has methodological limitations. The search was restricted to PubMed/MEDLINE and English-language material; Embase, Scopus, and Web of Science were not searched. The review did not use a prospectively archived MeSH-based strategy, independent duplicate screening, a PRISMA screening log, or formal risk-of-bias assessment. Screening counts could not be reconstructed reliably. These choices may have introduced selection, language, and publication bias.

Limitations also arise from the evidence base. Most SCA-specific publications are case reports, small series, or descriptive imaging studies with heterogeneous terminology, measurement methods, and follow-up. AAOCA cohorts and professional guidance provide indirect context for proximal morphology, functional testing, surgery, and exercise, but do not constitute SCA-specific outcome evidence. These limitations preclude quantitative synthesis and comparative management conclusions.

## 11. Conclusions

SCA is anatomically heterogeneous and should not be interpreted from a single-ostium label or Lipton code alone. In adults, detailed CCTA anatomy should be considered alongside symptoms, coexisting coronary disease, and selected mechanism-appropriate functional findings. The three-domain structure used here supports reporting and multidisciplinary discussion, but is not a validated risk model or treatment algorithm. At present, prospective SCA-specific outcome and management data remain insufficient.

## Figures and Tables

**Table 2:** Imaging modalities for anatomical definition, functional assessment, and diagnostic decision-making in SCA.

Modality	Primary Role	Strengths	Main Limitations	Practical Role and Evidence Boundary
Transthoracic echocardiography	Screening and ostial suspicion	Widely available; no radiation; useful especially in children and selected young patients.	Acoustic windows and incomplete course visualisation limit reliability in many adults.	Initial or complementary test. Complete adult SCA course mapping is often limited; supporting practice comes mainly from the broader anomaly and athlete screening literature [63].
Invasive coronary angiography	Lumen assessment and concomitant obstructive disease	High temporal resolution; familiar technique; useful when coronary disease or intervention is under consideration.	Two-dimensional projection can underrepresent complex three-dimensional relationships and a suspected intramural segment.	Important for luminal disease and intervention. Direct SCA PCI evidence is limited to a report and a small series [64,72]; a broader anomalous coronary PCI cohort provides indirect context [15]. Three-dimensional anatomy may remain uncertain without CCTA.
CCTA	Comprehensive anatomical mapping	Preferred non-invasive modality for detailed assessment of ostial origin, proximal course, spatial relationships, ostial morphology, and a suspected intramural segment.	Radiation and contrast exposure; functional significance cannot be assumed from anatomy alone.	Primary anatomical modality in many adults [18,43]. Selection depends on renal function, contrast, radiation, rhythm, heart rate, and image quality [18,67]; morphology alone does not establish functional significance [3,54].
CMR	Complementary structural and functional assessment	No ionising radiation; adds ventricular function, tissue characterisation, and selected perfusion information.	Usually less detailed than CCTA for fine coronary anatomy; availability and workflow vary.	Complementary for ventricular function, scar, tissue characterisation and selected perfusion questions; usually not the primary method for fine coronary mapping [16,18].
Intravascular ultrasound (IVUS)	Cross-sectional assessment of the ostium and proximal lumen	Directly demonstrates lumen shape and may show dynamic lateral compression during a defined manoeuvre.	Invasive and operator-dependent; direct SCA evidence is limited to an individual report and a small case series.	Practical invasive reference assessment of ostial and proximal lumen shape or dynamic compression. Direct SCA evidence is limited to a small case series and an individual report [45,55]; prospective cohort evidence is derived from R-AAOCA [69].
CT-derived fractional flow reserve (FFRCT)	Non-invasive physiological assessment derived from CCTA	Adds lesion-specific physiological information without an additional invasive procedure.	Model- and image-quality-dependent; SCA-specific validation and outcome thresholds are lacking.	May complement CCTA. Direct SCA evidence is limited to a multimodality case report [8]; SCA-specific validation and outcome thresholds are lacking. Cohort evidence is derived from broader AAOCA populations [70,71].
Exercise ECG and stress imaging	Symptom reproduction and physiological correlation	Exercise ECG, stress echocardiography, nuclear perfusion, and stress CMR provide different forms of physiological information.	Protocols and endpoints differ; fixed coronary disease and other non-obstructive mechanisms may complicate attribution in adults.	Exercise ECG and stress imaging answer different questions. Across these functional modalities, direct SCA evidence is limited to a case report and a small multimodality case series [8,45]; neither publication validates every test listed in this row. Comparative physiological evidence is derived from R-AAOCA [54].
Invasive physiological assessment (FFR)	Assessment of fixed or dynamic pressure loss using a defined provocation protocol	Can interrogate a specific proximal segment using adenosine or a dobutamine–atropine–volume challenge.	Invasive, protocol-dependent, and technically demanding; adenosine and dobutamine results are not equivalent.	Direct SCA evidence is limited to an individual report [55]. Comparative protocol data come from R-AAOCA [69] and adult AAOCA [60] cohorts and require cautious extrapolation.

Abbreviations: AAOCA, anomalous aortic origin of a coronary artery; CCTA, coronary computed tomography angiography; CMR, cardiac magnetic resonance imaging; ECG, electrocardiography; FFR, fractional flow reserve; FFRCT, CT-derived fractional flow reserve; IVUS, intravascular ultrasound; PCI, percutaneous coronary intervention; R-AAOCA, anomalous aortic origin of the right coronary artery; SCA, single coronary artery. The modalities answer different anatomical and physiological questions and should not be treated as equivalent sources of evidence.

**Table 3:** Representative adult SCA scenarios, priority questions, and evidence boundaries.

Adult Scenario	Priority Question	Priority Information	Interpretation, Competing Explanation, Procedural Concern, and Evidence Boundary
Incidental SCA without symptoms or plaque	Is there a proximal or branch-level feature that changes interpretation?	Complete CCTA anatomy and baseline clinical context	Lipton et al. reported an angiographic frequency of 0.024% [6], and Desmet et al. reported 0.066% [7]. CCTA referral cohorts reported different frequencies [41,43]. In larger angiographic datasets, Türkmen et al. reported 0.031% [51], while Akcay et al. identified 10 cases among 70,850 angiograms [52]. Detection alone does not establish a need for treatment.
Interarterial or suspected intramural course without demonstrable ischaemia	Did the test reproduce the suspected dynamic mechanism?	Ostium, proximal narrowing, intramurality, and the quality and protocol of functional testing	Mechanistic interpretation is derived mainly from AAOCA morphology studies [3,11]. Invasive functional evidence and documented test discordance also come from AAOCA cohorts [60,69], while recommendations are AAOCA-specific [73]. No SCA-specific outcome threshold is available.
Obstructive disease in a distal branch	Does the fixed stenosis explain the symptoms or ischaemia?	Lesion severity, supplied territory, and lesion-specific physiology when indicated	Acquired disease may coexist with SCA. Direct evidence includes small adult SCA and PCI series [13,64]; the anomaly itself should not be assumed to cause the plaque.
Sole-ostial or true common trunk stenosis	How much myocardium depends on the diseased segment?	Lesion location relative to branch points, trunk calibre and length, plaque burden, and distal targets	A lesion before the major branches separate may be left-main-equivalent [53]. PCI evidence is limited to reports and a small SCA series [64,72]; CABG evidence includes an isolated case [74].
Acute coronary syndrome	How can the culprit lesion be reached without jeopardising the sole ostium or another dependent branch?	Ostial orientation, complete branch map, culprit lesion, and catheter strategy	Urgent treatment follows the acute presentation. Direct SCA reports describe catheter selection and guide support [64,72]; a broader anomalous coronary PCI cohort provides indirect procedural context [15].
Symptoms without obstructive coronary disease	Is SCA causal, incidental, or one of several possible mechanisms?	Detailed anatomy, mechanism-matched stress findings, and targeted assessment of alternative cardiac causes when indicated	Symptoms should not be attributed automatically to SCA. INOCA and other cardiac causes remain competing explanations [56].
SCA identified before valve or aortic surgery	Could the operation jeopardise the sole inflow or a dependent branch?	Three-dimensional relationship to the annulus and aortic root, trunk length, branch course, and planned method of coronary protection	The ostium and all dependent branches require explicit protection. Technique is individualised and supported mainly by case evidence [14,75].
Concordance or discordance	Do anatomy, physiology, and symptoms indicate the same mechanism?	Test quality and timing, alternative causes, and the purpose of the assessment	Concordance strengthens a causal interpretation; discordance identifies uncertainty and may justify focused reassessment. Discordance across modalities is illustrated in AAOCA evidence [60,61]. This row is an organisational aid, not a validated score.

Abbreviations: AAOCA, anomalous aortic origin of a coronary artery; CABG, coronary artery bypass grafting; CCTA, coronary computed tomography angiography; INOCA, ischaemia with non-obstructive coronary arteries; PCI, percutaneous coronary intervention; SCA, single coronary artery. Evidence ranges from direct SCA case reports and small series to AAOCA cohorts and consensus guidance; these sources are not treated as equivalent.

## Data Availability

No new data were created or analysed in this study. Data sharing is not applicable to this article.

## Supplementary Materials

The following supporting information can be downloaded at https://www.mdpi.com/article/10.3390/jcm15166435/s1. Table S1: Principal SCA-specific studies informing classification, prevalence, multimodality imaging, and adult clinical/procedural interpretation.
