# Supplementary material for: Single Coronary Artery in the Context of Vascular Disease: Anatomy, Development, Multimodality Imaging, and Clinical Interpretation
Source: J Clin Med. 2026 Aug 20;15(16):6435. doi: 10.3390/jcm15166435 (PMC13513484; doi:10.3390/jcm15166435)
Supplement: Supplementary file 1 [file jcm-15-06435-s001.zip › jcm-4480993-supplementary.pdf]

SUPPLEMENTARY MATERIAL

# Single Coronary Artery in the Context of Vascular Disease: Anatomy, Development, Multimodality Imaging, and Clinical Interpretation

**Supplementary Table S1:** Principal SCA-specific studies informing classification, prevalence, multimodality imaging, and adult clinical/procedural interpretation.

Scope and purpose of the evidence map: Studies were selected for this focused narrative evidence map because they directly informed a review domain—classification, prevalence, coronary computed tomography angiography (CCTA)-based anatomy, or the adult clinical/procedural context—and because their design, sample, and evidence boundary can be stated transparently. The table is selective and non-exhaustive; it is not a systematic study selection table, quality ranking, or pooled analysis.

| Study and evidence domain | Design and sample | SCA phenotype / anatomical focus | Main method(s) | Principal contribution to this review | Evidence boundary |
|---|---|---|---|---|---|
| Lipton et al. (1979) [6] Foundational classification | Angiographic case series; 10 patients with isolated SCA. | Sinus of origin (R/L), distribution groups I–III, and proximal-course categories A/B/P. | Invasive coronary angiography and anatomical classification. | Established the most widely used coding framework for SCA origin, distribution, and proximal course. | Small historical series from the pre-CCTA era; the classification is descriptive and was not developed as a risk or management model. |
| Shirani and Roberts (1993) [35] Foundational anatomy | Ten necropsy cases plus review of 87 previously reported cases (35 necropsy; 52 angiographic). | Location of the solitary ostium, presence of an aberrant artery, and the route taken by that artery; 20 anatomical categories. | Necropsy examination combined with a historical literature review. | Provided a detailed complementary classification and emphasised clinically and surgically relevant distribution patterns. | Historical pooled evidence vulnerable to publication, referral, and duplicate case bias; not a prospective or contemporary imaging cohort. |
| Desmet et al. (1992) [7] Angiographic prevalence | Review of 50,000 consecutive adult coronary angiograms; 33 SCA cases (0.066%). | True single-ostium anatomy and angiographic subtype distribution. | Search of angiographic reports followed by review and classification of the corresponding films. | Provides a primary angiography-based SCA detection frequency and adult clinical and anatomical context. | Catheterisation-selected adult population and historical imaging; the estimate is not general population prevalence. |
| Akcay et al. (2008) [52] Adult angiographic follow-up | Retrospective review of 70,850 angiograms; 10 adult SCA cases; mean reported follow-up 54 ± 14 months. | Side of origin, angiographic pattern, coexisting atherosclerosis, and management. | Coronary angiography with retrospective clinical follow-up. | Adds descriptive middle-aged/older-adult follow-up and illustrates the coexistence of SCA and acquired CAD. | Only 10 cases and no comparator. The published 0.024% conflicts with 10/70,850 (approximately 0.014%); raw counts should be used rather than the reported percentage. Management adequacy cannot be inferred. |
| Türkmen et al. (2014) [51] Angiographic prevalence | Retrospective review of 215,140 routine coronary angiograms; 67 SCA cases (0.031%). | Right- versus left-sided origin and Smith/Lipton angiographic subtype distribution. | Invasive coronary angiography with retrospective classification. | Largest direct SCA-specific angiographic series included; supports detection frequency and subtype distribution. | Single-centre, catheterisation-selected population; retrospective ascertainment and limited outcome information. |

**Supplementary Table S1. Continued**

| Study and evidence domain | Design and sample | SCA phenotype / anatomical focus | Main method(s) | Principal contribution to this review | Evidence boundary |
|---|---|---|---|---|---|
| Mandal et al. (2014) [42]<br>Cross-sectional imaging | Retrospective imaging series of 22 adult and paediatric patients with SCA. | Interarterial, retroaortic, prepulmonic, septal, combined, and other SCA courses; associated CHD and CAD. | Multidetector computed tomography and magnetic resonance angiography retrieved from an imaging archive. | Demonstrated the value of cross-sectional imaging for three-dimensional course characterisation beyond catheter angiography. | Mixed paediatric/adult population; 9 patients had coexisting CHD; retrospective and heterogeneous imaging with no standardised longitudinal outcome comparison. |
| Michalowska et al. (2019) [41]<br>CCTA prevalence and morphometry | Screening of 30,230 CCTA examinations; 17 SCA cases (0.056%) compared with 199 controls with normal coronary anatomy. | SCA origin/course and proximal common trunk length, lumen area, and diameter. | Retrospective CCTA identification with quantitative morphometric comparison. | Combines CCTA-based detection frequency with controlled proximal trunk morphometry. | Only 17 SCA cases; retrospective referral population; anatomical measurements do not establish haemodynamic significance or prognosis. |
| Al Umairi and Al-Khouri (2019) [43]<br>CCTA spectrum and follow-up | Retrospective review of 4,445 consecutive CCTA examinations; 12 SCA cases (0.27%). | Modified Lipton subtype, course, associated CAD, and clinical management/outcome. | CCTA; selected invasive angiography and functional testing; descriptive follow-up. | Adds a defined CCTA denominator, detailed anatomical spectrum, coexisting CAD, and short-term clinical observations. | Single-centre and only 12 cases; the unusually high detection frequency should not be generalised. Follow-up duration is internally inconsistent (25.3 months in the abstract/table versus 23.3 months in the results text); absence of observed MACE is not evidence of safety. |
| Sheng et al. (2021) [45]<br>Multimodality anatomy and physiology | Five-patient SCA case series with varying Lipton group I/II courses. | Prepulmonic, transseptal/subpulmonic, and interarterial patterns and their possible functional relevance. | CCTA, CMR, invasive angiography, nuclear testing, and selected iFR and IVUS measurements at rest and during pharmacological stress. | Illustrates how anatomy, symptoms, and selected physiological testing can be integrated in individualised SCA assessment. | Highly selected case series with nonuniform testing and management; cannot establish diagnostic accuracy, treatment effectiveness, or comparative outcomes. |
| Said et al. (2014) [13]<br>Adult CAD and management | Descriptive multicentre series; 15 symptomatic adults with isolated SCA from four Dutch angiography centres. | SCA origin/course, concomitant CAD, ischaemic evaluation, and medical, PCI, or CABG management. | Coronary angiography in all patients; multislice computed tomography and functional tests in selected patients; clinical management review. | Provides direct adult SCA evidence at the interface of anomalous anatomy, acquired CAD, symptoms, and revascularisation planning. | Small, symptomatic, referral-selected series with heterogeneous testing; treatment choices and outcomes are descriptive and do not support causal or comparative management claims. |
| Vijayvergiya et al. (2021) [64]<br>PCI and procedural context | Fifteen-year retrospective catheterisation review: 12 SCA patients; 9 underwent 11 successful PCI procedures. | SCA course plus left-main, bifurcation, chronic total occlusion, calcified, and multivessel CAD interventions. | Angiography, modified Lipton classification, CT/course assessment in selected cases, PCI, IVUS, and rotational atherectomy. | Most focused direct SCA PCI series included; documents technical feasibility and procedural planning in complex acquired CAD. | Single-centre, retrospective, very small procedural series; technical success does not establish comparative safety, long-term effectiveness, or superiority of PCI over surgery/medical therapy. |

**Interpretive notes.** Detection frequencies are specific to the angiography or CCTA populations examined and should not be interpreted as general-population prevalence. Observations from uncontrolled series do not constitute comparative effectiveness, safety, or treatment-guideline evidence.

**Scope boundary of Table S1.** Where relevant, the main-text narrative also draws on broad coronary artery anomaly (CAA) and anomalous aortic origin of a coronary artery (AAOCA) cohorts, secondary reviews, pictorial reviews, and isolated illustrative case reports. These sources were not used to populate this focused evidence map of principal studies.

**Abbreviations.** AAOCA, anomalous aortic origin of a coronary artery; CAD, coronary artery disease; CAA, coronary artery anomaly; CABG, coronary artery bypass grafting; CCTA, coronary computed tomography angiography; CHD, congenital heart disease; CMR, cardiac magnetic resonance imaging;CT, computed tomography; iFR, instantaneous wave-free ratio; IVUS, intravascular ultrasound; MACE, major adverse cardiovascular event; PCI, percutaneous coronary intervention;R/L, right/left coronary sinus; SCA, single coronary artery. Reference numbers correspond to the 85-item reference list in the main manuscript.
